# Polymorphisms in the Tumor Necrosis Factor Receptor Genes Affect the Expression Levels of Membrane-Bound Type I and Type II Receptors

**DOI:** 10.1155/2014/745909

**Published:** 2014-03-24

**Authors:** Sergey V. Sennikov, Filipp F. Vasilyev, Julia A. Lopatnikova, Nadezhda S. Shkaruba, Alexander N. Silkov

**Affiliations:** Laboratory of Molecular Immunology, Federal State Budgetary Institution “Research Institute of Clinical Immunology”, Russian Academy of Medical Sciences Siberian Branch, 14, Yadrintsevskaya Street, Novosibirsk 630099, Russia

## Abstract

The level of TNF receptors on various cells of immune system and its association with the gene polymorphism were investigated. Determining the levels of membrane-bound TNF**α** receptors on peripheral blood mononuclear cells (PBMCs) was performed by flow cytometry using BD QuantiBRITE calibration particles. Soluble TNF**α** receptor (sTNFRs) levels were determined by ELISA and genotyping was determined by PCR-RFLP. Homozygous TT individuals at SNP −609G/T TNFRI (rs4149570) showed lower levels of sTNFRI compared to GG genotype carriers. Homozygous carriers of CC genotype at SNP −1207G/C TNFRI (rs4149569) had lower expression densities of membrane-bound TNFRI on intact CD14^+^ monocytes compared to individuals with the GC genotype. The frequency differences in the CD3^+^ and CD19^+^ cells expressing TNFRII in relation to SNP −1709A/T *TNFRII* (rs652625) in healthy individuals were also determined. The genotype CC in SNP −3609C/T *TNFRII* (rs590368) was associated with a lower percentage of CD14^+^ cells expressing TNFRII compared to individuals with the CT genotype. Patients with rheumatoid arthritis had no significant changes in the frequencies of genotypes. Reduced frequency was identified for the combination TNFRI −609GT + TNFRII −3609CC only. The polymorphisms in genes represent one of cell type-specific mechanisms affecting the expression levels of membrane-bound TNF**α** receptors and TNF**α**-mediated signaling.

## 1. Introduction

Tumor necrosis factor (TNF*α*) is a pleiotropic cytokine that plays an important role in mediating various immune functions including inflammation [1, 2], the regulation of apoptosis and necrosis [3], and induction of cytotoxicity [4]. TNF*α* is capable of eliciting a variety of different immune responses by signalling via two types of membrane-bound receptors, type I (CD120a, TNFRSF1A) and type II (CD120b, TNFRSF1B) receptors, with respective molecular weights of 55 and 75 kDa [5, 6]. Type I TNF*α* receptors (TNFRI) are more widespread and expressed on all cell types in contrast to type II TNF*α* receptors (TNFRII) expressed mainly on cells of the immune system [6, 7].

TNFRI are activated via both soluble and membrane-bound (mTNF*α*) forms of tumor necrosis factor alpha (TNF*α*), whereas TNFRII are mainly activated by mTNF*α* [8]. Most biological effects of TNF*α* (such as cytotoxicity and proliferation) are realized via TNFRI activation [6]. The intracellular TNFRI domains, in contrast to the intracellular domains of TNFRII, contain a death domain (DD) associated with TNF-mediated cytotoxicity [9]. The main function of TNFRII is proliferation induction in addition to apoptosis induction via a DD-independent mechanism [10]. There also exist two soluble TNF*α* receptor forms [11] generated by proteolysis of membrane-bound receptors [12, 13] or alternative splicing [14] that play an important role in TNF*α*-mediated biological activity [15]. Soluble TNF*α* receptors (sTNFR) do not allow binding to membrane-bound receptors thereby inhibiting TNF*α* biological activity [16].

The* TNFRI* gene is located on chromosome 12p13 consisting of 10 exons [17, 18] and contains a housekeeping promoter with multiple transcription start sites, a high GC content, and missing consensus TATA and CAAT box motifs [19]. The* TNFRII* gene is located on chromosome 1p36 and also contains 10 exons [17, 20], a* TNFRII* promoter also high in GC content, but containing several consensus TATA box motifs [21].

What impact cytokines have on the nature of the developing immune response depends both on the percentage of cells expressing membrane-bound receptors and on receptor expression levels on respective cells [22]. Differences in cytokine receptor expression levels can be affected by receptor gene polymorphisms. Single nucleotide polymorphisms (SNPs) occurring in promoter regions upstream of genes may potentially affect the process of transcription [23–25]. SNPs have important influence on mRNA stability and translational efficiency and may influence susceptibility to many common diseases [25–28].

The aim of this study was to establish associations between polymorphisms in the TNF*α* receptor genes and membrane-bound type I and type II TNF*α* receptor expression levels on various mononuclear cell populations and to determine the levels of sTNFRs in the serum of healthy individuals.

## 2. Material and Methods

### 2.1. Study Population

Whole blood samples were obtained from the Blood Procurement Station Number 1 of the “Novosibirsk Blood Center” and sampling was carried out from a population (*n* = 150 healthy individuals) between the ages of 19 and 55 years from the city of Novosibirsk (South-Western Siberia). The main exclusion criteria were standard for blood donors in the Russian Federation. Also, 466 patients with rheumatoid arthritis (RA) were included in the study, of whom 86.5% were women and 13.5% were males, aged 18 to 70 years. The diagnosis was verified according to the ACR criteria. Research was performed in accordance with* The Code of Ethics of the World Medical Association* (Declaration of Helsinki) and was approved by the local ethics committee of the FSBI “Research Institute of Clinical Immunology”. All individuals provided informed consent before the study was carried out.

### 2.2. Measurement of Serum TNF*α* Levels and Soluble Types I and II TNF*α* Receptors

TNF*α* serum levels and the level of soluble types I and II TNF*α* receptors were determined. Soluble receptor levels were determined using enzyme-linked immunosorbent assay (ELISA) kits. Specifically, the human sTNF RI ELISA Kit and the human sTNF RII ELISA Kit (RayBiotech, Norcross, GA, USA) were used according to the manufacturer's instructions. TNF*α* levels were determined using the *α*-TNF-EIA-BEST (JSC Vector-Best, Novosibirsk, Russia).

### 2.3. Isolation and Culture of Peripheral Blood Mononuclear Cells (PBMCs)

PBMCs were isolated from the blood of healthy individuals using a standard Ficoll-Urografin density gradient method (*ρ* = 1.077 g/cm^3^) [29]. PBMCs were cultured at a concentration of 2 × 10^6^/mL in 96-well flat-bottom plates (TPP, Trasadingen, Switzerland) in the absence or presence of lipopolysaccharide (LPS) from* Escherichia coli* serotype 055:B5 (Sigma-Aldrich, St. Louis, MO, USA) at a final concentration of 200 ng/mL [30]. Cells were cultured in RPMI-1640 medium containing 10% fetal calf serum, 2 mM L-glutamine, 10 mM HEPES buffer, 0.5 mM 2-mercaptoethanol, 80 *μ*g/mL gentamicin, and 100 *μ*g/mL benzylpenicillin for 24 h at 5% CO_2_ and 37°C.

### 2.4. Determination of Membrane-Bound of TNF*α* Types I and II Receptor Levels

The number of cells expressing membrane-bound types I and II TNF receptors was determined by flow cytometry as described previously [31]. The antibodies labeled with phycoerythrin (PE) were used: anti-human TNF RI (R&D systems, Minneapolis, MN, USA, cat number FAB225P, clone 16803.1, mouse IgG1) and anti-human TNF RII (R&D systems, cat number FAB226P, clone 22235, mouse IgG2A). The following antibodies from eBioscience (San Diego, CA, USA) were used for immunophenotyping PBMC subpopulations: allophycocyanin (APC-) labeled anti-CD3 (cat number 17-0037, clone OKT3, mouse IgG_2A_), fluorescein isothiocyanate (FITC-) labeled anti-CD14 (cat number 11-0149, clone 61D3, mouse IgG_1_), and phycoerythrin-cyanine 7 (PE-Cy7) anti-CD19 (cat number 25-0199, clone HIB19, mouse IgG_1_).

To obtain the calibration curve and convert the fluorescence intensity of cells expressing corresponding markers to absolute receptors numbers, BD QuantiBRITE calibration particles (BD Biosciences, San Jose, CA, USA) were used. Flow cytometric analysis was performed using a BD FACSAria flow cytometer (BD Biosciences). We gated the populations for analysis on the basis of indices of forward (FSC-A) and side (SSC-A) scattering that were situated in the lymphocytic and monocytic regions. Subsequently, we selected subpopulations (CD3^+^ T lymphocytes, CD19^+^ B lymphocytes, CD14^+^ monocytes) on the basis of the presence of markers of these subpopulations. Further, we established an interval gate on the control histogram, which was obtained with samples incubated in the absence of anti-human TNFRI and TNFRII antibodies, and determined percent of positive events and mean fluorescence of cells expressing membrane-bound receptors for each of these subpopulations on PE/count histograms.

### 2.5. Genotyping Methods

Genomic DNA was isolated from PBMCs harvested from healthy individuals using phenol-chloroform extraction methods. SNPs selected for analysis for their association with receptor expression levels were selected from the NCBI dbSNP (http://www.ncbi.nlm.nih.gov/snp). SNP selection criteria were location within the promoter regions of the types I and II TNF receptor genes and high minor allele frequency (MAF) and by existence of associations with pathology. Additionally, SNPs were tested for the presence of transcription factor binding sites using software AliBaba2.1 (http://www.gene-regulation.com/pub/programs/alibaba2/index.html).

Genotyping polymorphisms at* TNFRI* −609G/T (rs4149570),* TNFRI* −1207C/G (rs4149569),* TNFRII* −1709A/T (rs652625), and* TNFRII* −3609C/T (rs590368) were conducted by polymerase chain reaction (PCR) in combination with RFLP (restriction fragment length polymorphism) analysis. Sequences of primers specific for SNPs* TNFRI* −609G/T,* TNFRI* −1207C/G, and* TNFRII* −1709A/T were described previously [32, 33]. Sequences of primers specific for* TNFRII* −3609C/T were designed with the aid of the NCBI/Primer-BLAST program (http://www.ncbi.nlm.nih.gov/tools/primer-blast). Primers specific for* TNFRI* and* TNFRII* gene sequences were synthesized by BIOSAN (Novosibirsk, Russia) (Table 1).

PCR was carried out using a PTC-200 DNA thermocycler (MJ Research Inc., Watertown, MA, USA). The 20 *μ*L reaction volume contained 1-2 units* Taq *DNA polymerase (SibEnzyme, Novosibirsk, Russia), 0.5 *μ*M of each primer, 0.25 mM of each desoxynucleoside-triphosphate, and 50–200 ng of genomic DNA. Reaction buffer was added to the DNA polymerase containing 60 mM Tris-HCl (pH 8.5, 25°C), 1.5 mM MgCl_2_, 25 mM KCl, 10 mM 2-mercaptoethanol, and 0.1% Triton X-100. PCR conditions were as follows: initial denaturation at 95°C for 3 min followed by 30 cycles for* TNFRI* −609G/T and* TNFRII* −1709A/T or 35 cycles for* TNFRI* −1207C/G and* TNFRII* −3609C/T at 94°C for 20 s; 61°C for 15 s (*TNFRI* −609G/T) or 58°C for 15 s (*TNFRI* −1207C/G) or 64°C for 15 s (*TNFRII* −1709A/T) or 63°C for 15 s (*TNFRII* −3609C/T); 72°C for 20 s, and a final extension at 72°C for 2 min.

Amplification products were exposed to respective restriction enzymes (5–10 activity units) in a volume 2.5–5 *μ*L (SibEnzyme). Restriction digestion of amplification products was carried out overnight at a temperature of 65°C for* TNFRI* −609G/T, 55°C for* TNFRI* −1207C/G, and 37°C for* TNFRII* −1709A/T and* TNFRII* −3609C/T.

Restriction products were analyzed by capillary electrophoresis using the QIAxcel System (Qiagen, Hilden, Germany) or 2% agarose gel electrophoresis at a voltage of 140–150 V for 20–25 min. QX DNA markers (Qiagen, Valencia, CA) and the pUC19 plasmid digested with Msp I (SibEnzyme) were used as molecular weight markers. Agarose gel electrophoresis visualized using the video densitometer ImageMaster VDS (Pharmacia Biotech).

### 2.6. Statistical Analysis

Data are expressed as the median and interquartile ranges. Phenotype frequency distribution with Hardy-Weinberg equilibrium was established using the *χ* ^2^ test. Correlation analyses were performed using the Spearman's rank correlation test. The relationship of the respective genotypes with TNF*α* receptor expression levels was tested using the Kruskall-Wallis ANOVA *H* test, Mann-Whitney *U* test, and the median test. A *P* value of ≤0.05 was considered statistically significant.

## 3. Results

### 3.1. Serum TNF*α* and Soluble Types I and II TNF*α* Receptor Levels

The TNF*α* and soluble TNF*α* types I and II receptor levels in the serum of 150 healthy individuals were determined. These experiments demonstrated that serum levels of soluble TNF*α* receptor type II (2449.9 [1915.1–3768.9] pg/mL) were significantly higher than those of soluble TNF*α* receptor type I (707.9 [497.8–939.9] pg/mL) (*P* < 0.001). This analysis also demonstrated that serum levels of sTNFRI in healthy individuals positively correlated with serum TNF*α* levels (0.669 [0–1.9] pg/mL) (*R* = 0.32, *P* < 0.05). The levels of TNF*α* negatively correlated with the absolute number of TNFRI expressed on CD3^+^ T cells and CD19^+^ B cells (*R* = −0.39  *и* *R* = −0.22, resp., *P* < 0.05).

### 3.2. Measurement of Membrane-Bound Types I and II TNF*α* Receptors

We observed differences in the expression levels of membrane-bound TNF*α* receptors on certain subpopulations of mononuclear cells, which may be indicative of different effector profiles of different immunocompetent cells in response to TNF*α*. These potentially different responses are affected by the percentage of TNFR positive cells in the context of the absolute number of TNF*α* receptors (Table 2). Difference in receptor level expression may be both due to expression differences by different mononuclear cell populations or due to TNF*α* receptor gene polymorphisms.

### 3.3. Genotyping Frequencies of the Study Population

TNF*α* receptor allele and genotype frequencies at the −609G/T and −1207G/C* TNFRI *positions and the −1709A/T and −3609C/T* TNFRII* positions were studied in healthy inhabitants of Novosibirsk (Table 3). The genotype and allele frequencies of all four polymorphisms were consistent with HWE criteria (*P* > 0.05).

### 3.4. Association of TNF*α* Receptor Gene Polymorphisms with Expression Levels of Membrane-Bound Receptors and Serum Levels of TNF*α* and Soluble Receptors

**Figure 1:**
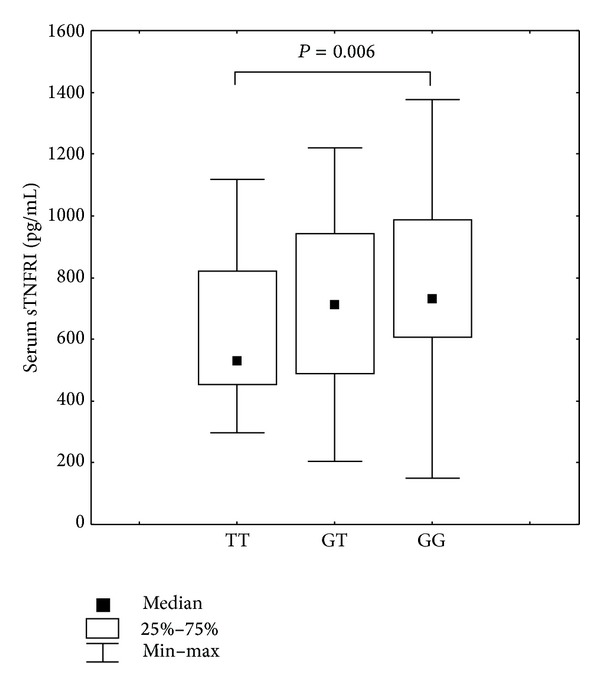
Serum levels of soluble TNF*α* type I receptor from individuals presenting with different SNP* TNFRI* −609G/T (rs4149570) polymorphisms. Kruskall-Wallis *H* test, *P* = 0.032, Mann-Whitney *U* test: TT versus GG, *P* = 0.006.

We did not observe associations between SNPs present in the promotor region of TNF*α* receptor genes and serum levels of TNF*α* and sTNFRII. When analyzing data regarding serum concentrations of soluble TNF*α* receptors and respective genotypes, we observed that individuals homozygous at the T allele at position −609G/T* TNFRI* (rs4149570) presented with lower levels of soluble TNF*α* receptor type I compared to individuals presenting with the G allele (Mann-Whitney *U* test, TT versus GG, *P* = 0.006; Kruskall-Wallis *H* test, *P* = 0.032) (Figure 1). The comparison of genotype frequencies at position −609G/T was also statistically significant with regard to differences in the percentage of CD19^+^ cells expressing membrane-bound TNFRI (Median test, *χ* ^2^ = 5.992, *P* = 0.05).

**Figure 2:**
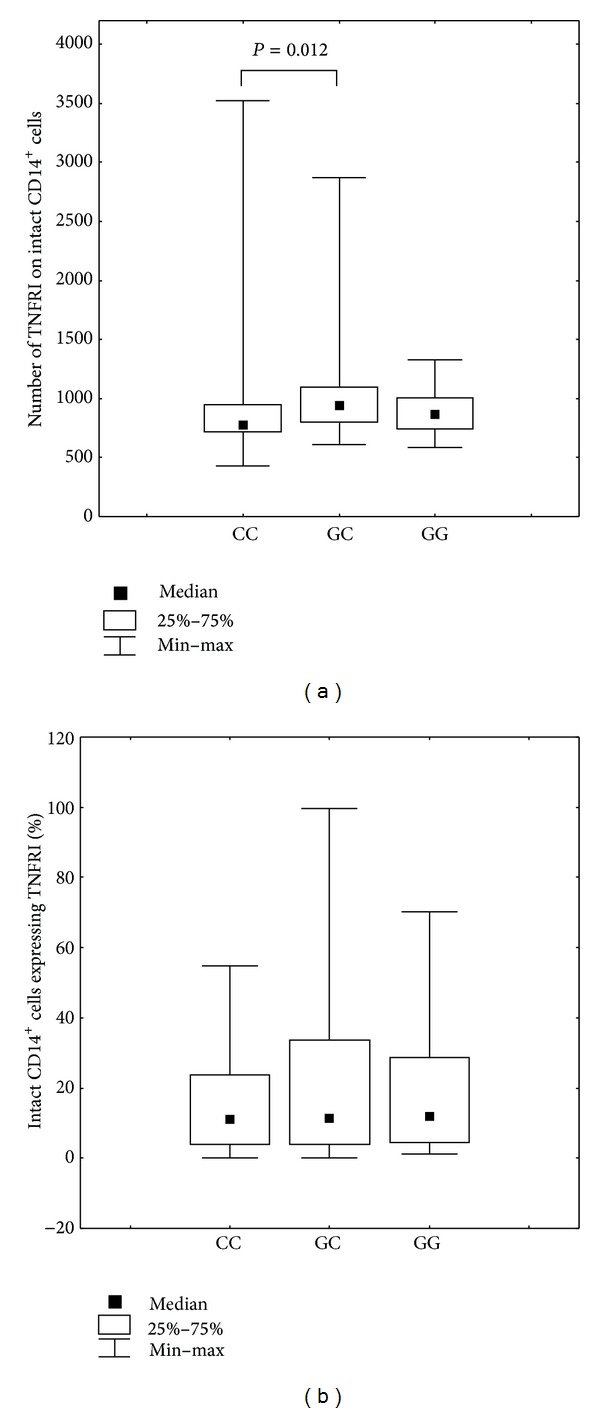
(a) Number of membrane-bound TNFRI present on CD14^+^ monocytes in individuals presenting with different SNP* TNFRI* −1207G/C (rs4149569) genotypes. Kruskal-Wallis *H* test, *P* = 0.025, Mann-Whitney *U* test: CC versus GC, *P* = 0.012. (b) Percentage of CD14^+^ cells expressing TNFRI in individuals presenting with different SNP* TNFRI* −1207G/C (rs4149569) genotypes.

The association between the expression level of membrane-bound TNF*α* receptor type I and genotype was established for SNP −1207G/C* TNFRI *(rs4149569). The homozygous CC genotype was statistically more frequent in the group with lower densities of CD14^+^ monocytes expressing surface TNFRI (Mann-Whitney *U* test, CC versus GC, *P* = 0.012; Kruskall-Wallis *H* test, *P* = 0.025; Median test, *χ* ^2^ = 7.325, *P* = 0.025) (Figure 2). We also demonstrated that frequencies in the genotypes of SNP −1207G/C were associated with different stimulation index values (Median test, *χ* ^2^ = 6.283, *P* = 0.043). The stimulation index was calculated as a simple ratio of absolute number of TNFRI receptors on CD14^+^ cells in cultures with and without LPS stimulation.

When analyzing* TNFRII* genotype frequencies at SNP −1709A/T (rs652625) we observed a statistically significant difference in the percentage of CD3^+^ and CD19^+^ cells expressing TNFRII in healthy individuals (Median test, *χ* ^2^ = 5.049, *P* = 0.024 and *χ* ^2^ = 4.560, *P* = 0.032, resp.).

**Figure 3:**
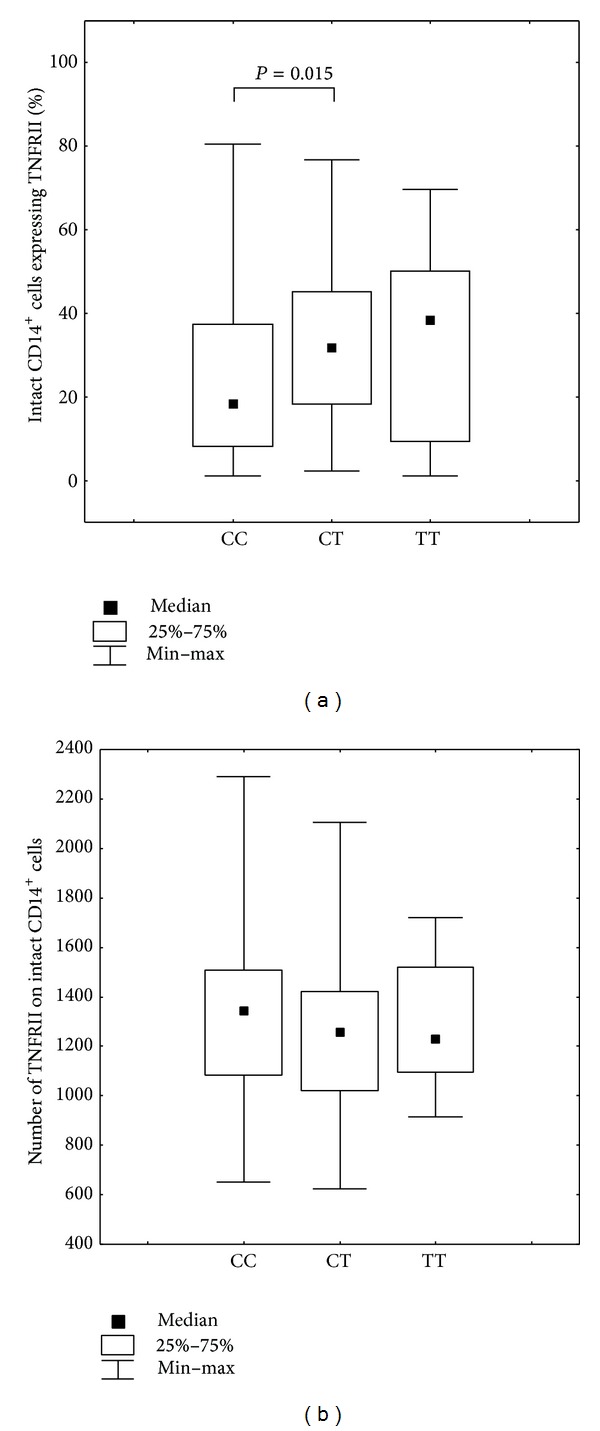
(a) Percentage of CD14^+^ cells expressing TNFRII in individuals presenting with different SNP* TNFRII* −3609C/T (rs590368) genotypes. Kruskall-Wallis *H* test, *P* = 0.041, Mann-Whitney *U* test: CC versus CT, *P* = 0.015. (b) Number of membrane-bound TNFRII present on CD14^+^ monocytes in individuals presenting with different SNP* TNFRII* −3609C/T (rs590368) genotypes.

Individuals with CC genotype at position −3609C/T (rs590368) of* TNFRII* had a lower percentage of intact CD14^+^ cells expressing TNFRII compared to individuals with the CT genotype (Mann-Whitney *U* test, CC versus CT, *P* = 0.015; Kruskall-Wallis *H* test, *P* = 0.041) (Figure 3).

### 3.5. Association of TNF*α* Receptor Gene Polymorphisms with Rheumatoid Arthritis

The frequencies of alleles and genotypes of TNFRI promoter at positions −609 and −1207 and TNFRII at positions −3609 and −1709 had no statistically significant differences in RA patients and healthy individuals. However, the analysis revealed a combination of genotypes TNFRI-609GT + TNFRII-3609CC. The frequency of this combination in patients was 10% and was significantly lower than that in the group of population controls 22% (*χ* ^2^ = 11.6, *P* = 0.0006). The Odds Ratio for this combination of genotypes was OR = 0,42 (CI95 = 0.25–0.71), and a relative risk of rheumatoid arthritis for carriers of this genotype was 10% lower. These combinations of genotypes comparative analysis are shown in Table 4.

**Figure 4:**
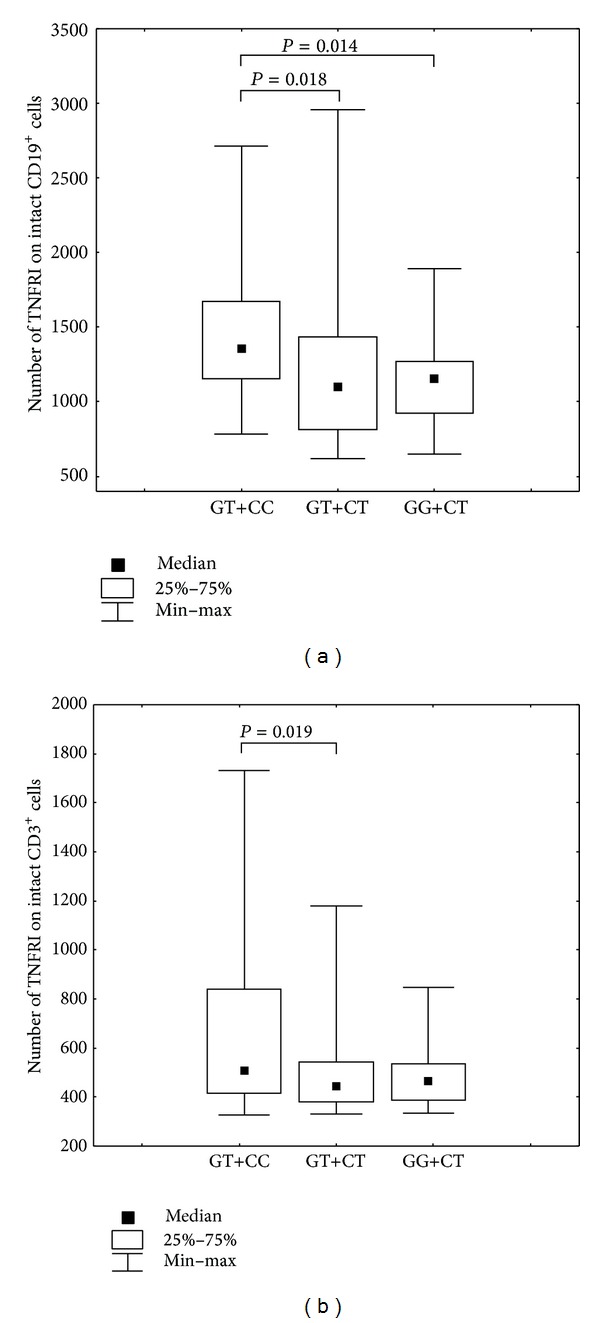
(a) Number of membrane-bound TNFRI present on CD19^+^ cells in individuals presenting with different genotypes combinations of SNPs* TNFRI* −609G/T (rs4149569) and* TNFRII* −3609C/T (rs590368). Kruskal-Wallis *H* test, *P* = 0.02, Mann-Whitney *U* test: GT+CC versus GT+CT, *P* = 0.02, GT+CC versus GG+CT, *P* = 0.014. (b) Number of membrane-bound TNFRI present on CD3^+^ cells in individuals with different genotypes combinations of SNPs* TNFRI* −609G/T (rs4149569) and* TNFRII* −3609C/T (rs590368). Kruskal-Wallis *H* test, *P* = 0.05, Mann-Whitney *U* test: GT+CC versus GT+CT, *P* = 0.02.

**Figure 5:**
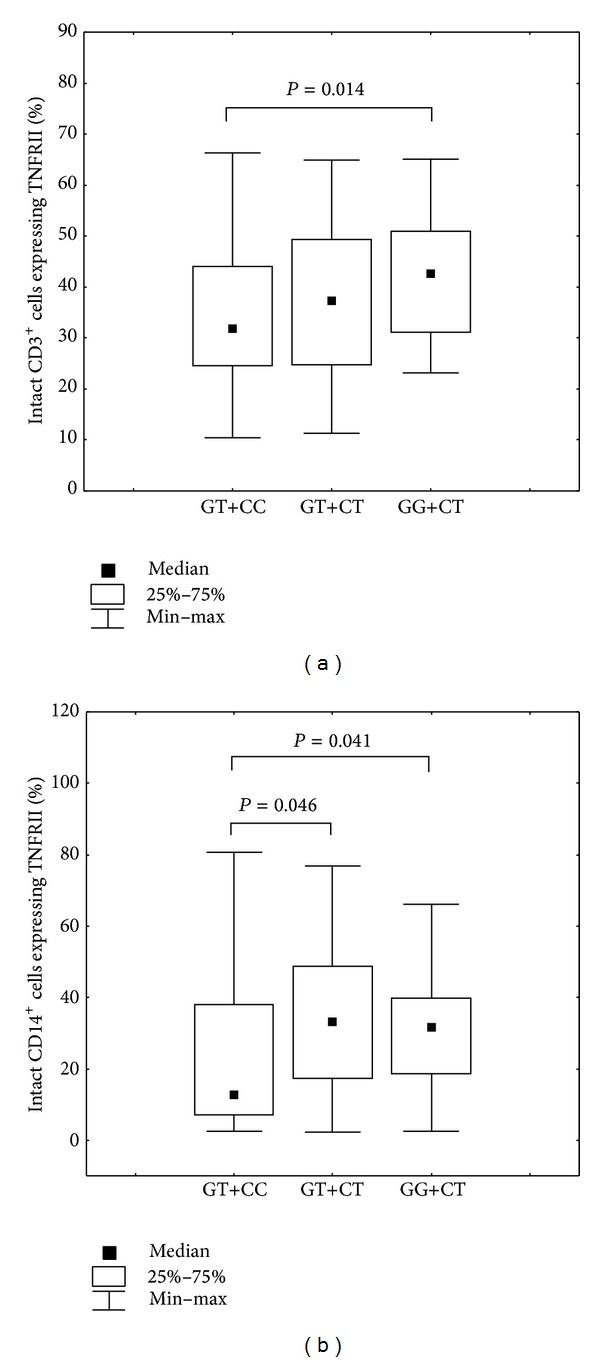
(a) Percentage of CD3^+^ cells expressing TNFRII in individuals presenting with different genotypes combinations of SNPs* TNFRI* −609G/T (rs4149569) and* TNFRII* −3609C/T (rs590368). Kruskal-Wallis *H* test, *P* = 0.048, Mann-Whitney *U* test: GT+CC versus GG+CT, *P* = 0.01. (b) Percentage of CD14^+^ cells expressing TNFRII in individuals with different genotypes combinations of SNPs* TNFRI* −609G/T (rs4149569) and* TNFRII* −3609C/T (rs590368). Mann-Whitney *U* test: GT+CC versus GT+CT, *P* = 0.046, GT+CC versus GG+CT, *P* = 0.04.

We have examined the association of combined genotypes with level of expression of TNF receptors in healthy donors. Individuals with the combination of GT+CC are characterized by an increase of membrane-bound TNFRI on intact subpopulations CD19^+^ B cells and CD3^+^ T lymphocytes (Figure 4) and reduced the percentage of CD3^+^ T lymphocytes and CD14^+^ monocytes expressing TNFRII (Figure 5). Serum levels of TNF*α* for combinations of genotypes had a trend to decrease in the series GG+CT-GT+CT-GT+CC. Data are not shown.

## 4. Discussion

Analyses of signaling mechanisms associated with TNF*α* are necessary to evaluate not only the cytokine itself and its soluble receptors but also membrane-bound receptors that confer different biological effects. It has been demonstrated that healthy individuals manifest quantitative differences in not only the percentage of cells expressing these receptors but also the quantity of receptors expressed. It can be inferred that different cell subpopulations would have different response to TNF*α* depending on receptor expression densities. It is probable that cells expressing a greater receptor density or if a cell population expresses a greater percentage of these receptors it would enhance the effects conferred by TNF*α* (on these cells). For this reason, the percentage of cells expressing TNF*α* receptors does not always correlate with the absolute number of receptors. For example, comparison of TNFRI expression by T and B lymphocytes and monocytes identified that CD19^+^ B lymphocytes expressed the lowest number of total TNFRI but as a population expressed the greatest density of receptors. By contrast, a greater percentage of CD3^+^ T lymphocytes expressed TNFRII at the lowest density of any cell type examined.

Previous work has demonstrated that cells cultured in the presence of LPS for 24 h resulted in a significant enhancement in TNRII expression compared to TNFRI expression in CD14^+^ monocytes [30]. Data presented in this report support these observations; that is, a higher percentage of monocytes cultured in the presence of LPS expressed TNFRII (at a higher density) compared to TNFRI expression (and density per cell) what testifies to a different involvement of TNF*α* receptors in response to LPS action. These data confirmed that LPS significantly affected TNFRII expression on CD14^+^ monocytes from healthy individuals. In addition, comparison of freshly isolated (unstimulated) CD14^+^ monocytes to Mock-stimulated CD14^+^ monocytes cultured for 24 h revealed differences both in the percentage of positive cells and in the expression level of membrane-bound TNF*α* receptors likely associated with microenvironment changes.

Analyses of correlation of TNF*α* with its soluble receptors have resulted in conflicting observations. For example, Spinas et al. [26] established a correlation between TNF*α* and sTNFRI levels but not with sTNFRII and Koga et al. [34] established a correlation between TNF*α* and sTNFRII but did not establish a correlation between TNF*α* and levels of sTNFRI. Data presented in this report demonstrated that serum TNF*α* levels positively correlated with sTNFRI levels in the serum of healthy individuals. We also demonstrated that serum sTNFRI (weakly) negatively correlated with that of the density of membrane-bound TNFRI expressed on cell surfaces, suggesting an association with proteolytically derived membrane-bound receptors. TNF*α* levels also negatively correlated with the levels of membrane-bound TNFRI on cells, supporting previous reports demonstrating that TNF*α* decreased in the amount of mRNA encoding for TNFRI [35].

Differences in the levels of receptor expression can also be affected by TNF*α* receptor gene polymorphisms. A considerable number of SNPs located within the promoter region of TNF-TNFR superfamily gens can affect regulation by significantly impacting levels of gene expression [36, 37]. The presence of certain alleles within promoter regions of cytokine receptor genes can influence gene transcription rates and mRNA stability resulting in increased or decreased levels of the synthesized protein. The SNPs analyzed during the course of this study were located within the TNF*α* receptor gene types I and II promoter regions and are therefore likely to affect TNFRs expression levels.

**Figure 6:**
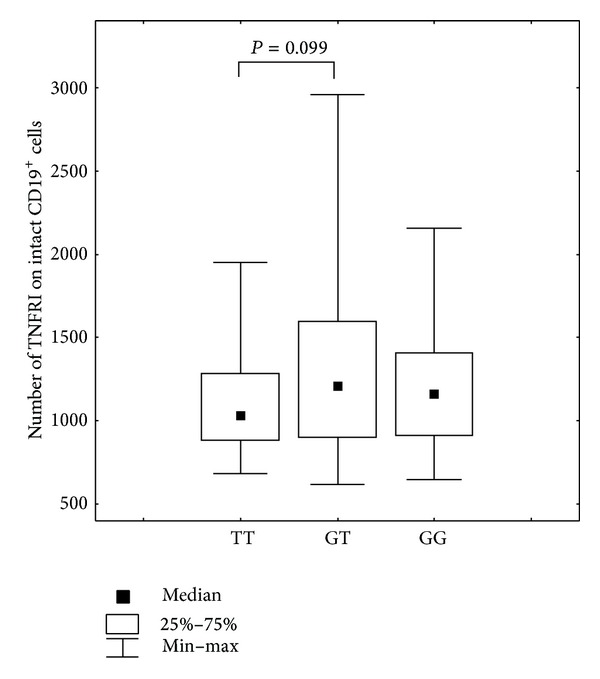
Number of membrane-bound TNF*α* type I receptor on CD19^+^ B cells in individuals presenting with different SNP* TNFRI* −609G/T (rs4149570) genotypes, Mann-Whitney *U* test: TT versus GT, *P* = 0.099.

Several studies have examined the association of polymorphisms at the* TNFRI* −609G/T (rs4149570) locus with various pathologies. For example, the T allele was significantly associated with systemic lupus erythematous [38], poor survival outcomes in non-small-cell lung cancers [39], and T cell non-Hodgkin's lymphoma [40]; however, this polymorphism was protective against oral carcinoma [41], which decreased the risk of colon cancer [42] and invasive pulmonary aspergillosis [43]. Kim et al. [44] found out that the* TNFRI* −609G/T polymorphism was strongly associated with primary hepatocellular carcinoma and that the T allele repressed TNFRI expression. The present study demonstrated that individuals homozygous for the T allele of SNP −609G/T located within the* TNFRI* gene promoter presented with lower serum levels of soluble type I TNF*α* receptors. It has been demonstrated that soluble receptors inhibit the biologic effects of TNF*α* [15]; therefore, when soluble receptors are present at lower concentrations there is less competition for membrane-bound receptors. A tendency has also been demonstrated to the lowering of the absolute numbers of membrane-bound TNFRI on intact CD19^+^ B cells in individuals with the TT genotype (Mann-Whitney *U* test, TT versus GT, *P* = 0.099) (Figure 6). Considering that soluble TNF*α* type I receptors are formed as by-products of proteolytic cleavage from membrane-bound TNF*α* receptors [12, 13], it can be concluded that the smaller amounts of soluble TNFRI associated with the TT genotype are directly associated with diminished expression levels of membrane TNFRI levels. Reduced expression of TNF*α* receptors appears to be associated with the G allele that encodes for the binding site of the interferon consensus sequence-binding protein (ICSBP, also known as IRF8 or interferon regulatory factor 8), a transcription factor that is involved in TNFRI-mediated activation of NF-*κ*B signaling pathway [43].

Miyagawa et al. [38] demonstrated that, for SNP* TNFRI* −1207G/C (rs4149569), the C allele frequencies in patients with systemic lupus erythematous were significantly lower than the frequencies in control groups. The present study demonstrated that CC genotype carriers at position −1207G/C of the* TNFRI* gene presented with a reduced density of TNFRI on CD14^+^ monocytes. It has been demonstrated using the online AliBaba2.1 (http://www.gene-regulation.com/pub/programs/alibaba2/index.html) program that this SNP (in the context of the C allele) was associated with lack of transcription factor binding sites and that the G allele was associated with transcription factor binding sites for C/EBPalpha (also known as CCAAT/enhancer-binding protein alpha), AP-2alpha (also known as TFAP2A), and Sp1. It is quite probable that the differences in expression of TNFRI on cells of individuals with different genotypes are associated with one of these transcription factors.

A number of studies have established an association between SNP* TNFRII* −1709A/T (rs652625) with pathology [45, 46]. Steenholdt et al. [47] determined that the A allele in SNP −1709A/T of the* TNFRII* gene increased the risk of severe infusion reactions to infliximab in Crohn's disease patients. We examined the frequency of allelic variants of* TNFRI* and* TNFRII* genes in patients with rheumatoid arthritis and demonstrated that RA patients (compared to controls) were significantly less likely to present with* TNFRI* −609GT +* TNFRII* −3609CC combination of genotypes. Individual's predisposition to developing of disease may be determined by the individual characteristics of the expression regulation of TNF-*α* and its receptors in the cells of the immune system. The present study identified statistically significant frequency differences in the percentage of CD3^+^ and CD19^+^ cells expressing TNFRII in individuals carrying AA genotype in SNP* TNFRII −*1709A/T (rs652625). Individuals homozygous for the C allele in SNP −3609C/T (rs590368) of the* TNFRII *gene had low percentage of CD14^+^ cells expressing TNFRII. Using AliBaba2.1 we demonstrated a difference at the binding site defined by the −1709A/T of* TNFRII* allele. Specifically, transcription factors did not bind to the sequence encoded by the T allele and the sequence encoded by the A allele resulting in CFT binding (also known as transcription factor NF-I). The biologic effects of TNF*α* result from interactions with two types of membrane-bound receptors: TNFRI and TNFRII. It is known that simultaneous expression of TNFRI and TNFRII results in the degradation of TRAF2 resulting in increased TNFRI-mediated cytotoxicity [10, 48]. These data suggested that signaling through TNFRI and TNFRII determined cellular survival. It is quite probable that cell populations expressing higher levels of TNFRII would be associated with higher rates of apoptosis.

Thus, we have established that SNPs −609G/T and −1207G/C of* TNFRI* gene promoter and −1709A/T and −3609C/T of* TNFRII* gene promoter are associated with expression level of TNF*α* receptors what specifies that these polymorphisms are functional. Association of SNPs −1207G/C, −1709A/T, and −3609C/T of* TNFR* genes promoters with expression levels of membrane-bound TNF*α* receptors types I and II in the absence of association with level of soluble TNF*α* receptors is established what testifies to existence of different mechanisms of regulation of soluble and membrane-bound receptors expression. Association of SNPs −1207C/T and −3609C/T with expression of TNFRs on CD14^+^ population in the absence of association with expression on CD3^+^ and CD19^+^ subpopulations testifies to a functional role of these SNPs for separate subpopulations of mononuclear cells. A possible mechanism for determining the expression of the receptor is a cell-specific transcriptional regulation of a set of factors (enhancers and repressors) [49, 50].

Interesting results were obtained in the analysis of combinations of genotypes. Combination TNFRI −609GT (rs4149569) and TNFRII −3609CC rarely is detected in RA patients and is associated with increased levels of TNFRI and reduced level of TNFRII on the immune cells. Perhaps different levels of TNF receptors types I and II on the cells determine the relationship of genetic variants with rheumatoid arthritis. However, for certain output, a broader study is necessary.

## 5. Conclusion

This study identified differences in the percentage of cells expressing TNF*α* receptors and in the absolute number of membrane-bound receptors expressed by PBMCs. Also we have established that the percentage of cells expressing TNFRs is not always associated with the absolute number of receptors. Furthermore, we determined that differences in expression levels of TNF*α* receptors types I and II could be associated with* TNFRI* and* TNFRII* gene polymorphisms. Associations of SNPs located within the promoter regions of TNF*α* type I and type II receptor genes were established in the context of expression levels of membrane-bound receptors present on subpopulations of mononuclear cells and with the serum levels of soluble type I TNF*α* receptors. These observations suggested that TNF*α* receptor gene alleles represent one of the factors that affects variability in the expression of membrane-bound receptors that may explain differences in the effects mediated by TNF*α* on different cell populations/subpopulations.

## Figures and Tables

**Table 1:** Primers and restriction endonucleases used for SNP genotyping.

SNP	Primers	Sequence (5′ to 3′)	Restriction endonucleases
*TNFRI* − 609 G/T, rs4149570	Forward Reverse	CGGACGCTTATCTAT ATCTC TTGTAGTCCAGTCACAAGCA	Bst4C I

*TNFRI* − 1207 C/G, rs4149569	Forward Reverse	TTGGGAGATGTCTGCATCAA TTCTTCGTTTGCTTGTTTTTCA	BstC8 I

*TNFRII* − 1709 A/T, rs652625	Forward Reverse	GAGTGCTGAGTGAGAAACTG AGCTTGAATTCGTTCCCAGG	DseD I

*TNFRII* − 3609 C/T, rs590368	Forward Reverse	ATGCTTTTGTCCATGCAGGT GCTGTACCCCGTATTAGCTG	Msp I

SNP: single nucleotide polymorphism; TNFR: tumor necrosis factor receptor.

**Table 2:** The level of membrane-bound TNF*α* types I and II receptors expressed on CD3^+^, CD19^+^, or CD14^+^ PBMC subpopulations.

	Percent of cells expressing receptors		Number of receptors per cell	
	TNFRI	TNFRII	TNFRI	TNFRII
CD19^+^ cells	1.3 (0.9–2.1)*	7.9 (5.8–12.3)*	1153.7 (891.9–1490.8)*	1102.5 (933.4–1309.2)*
CD3^+^ cells	1.5 (0.9–2.6)*	36.6 (28.4–47.6)**	427.3 (349.2–524.7)**	570.3 (516.8–627.1)**
CD14^+^ cells	11.4 (4.0–28.8)	28.3 (12.2–43.3)***	869.4 (756.9–1017.9)***	1273.9 (1053.0–1450.6)***
Mock-stimulated CD14^+^ cells^1^	9.8 (5.8–35.0)	50.0 (31.2–72.1)^†^	1267.5 (1053.0–1521.1)^†^	1983.4 (1677.9–2268.9)^†^
LPS-stimulated CD14^+^ cells^1^	9.6 (4.0–32.3)	79.2 (71.6–87.8)	1718.2 (1245.0–2559.1)	4177.0 (3133.1–5173.5)

Data are expressed as median values (interquartile range).

^1^Mononuclear cells (2 × 10^6^/mL) were cultured in the absence or presence of LPS (*E. coli* serotype 055:B5) at a concentration of 200 ng/mL for 24 h. *Significantly different from CD14^+^ cells (*P* < 0.001); **significantly different from CD19^+^ and CD14^+^ cells (*P* < 0.001); ***significantly different from CD14^+^ cells harvested from mock-stimulated PBMCs (*P* < 0.001); ^†^significantly different from CD14^+^ cells harvested from LPS-stimulated PBMCs (*P* < 0.001).

**Table 3:** Genotype and allele distributions of *TNFRI* and *TNFRII* gene polymorphisms from healthy individuals from Novosibirsk (South-Western Siberia, Russia, *n* = 150).

Gene	SNP	Genotype and allele	%	*n*	*P* ^a^
*TNFRI *	−609 G/T, rs4149570	GG	30.9	46	0.405
		GT	52.3	78	
		TT	16.8	25	
		G	57		
		T	43		

*TNFRI *	−1207 G/C, rs4149569	GG	34.7	52	0.942
		GC	48.7	73	
		CC	16.7	25	
		G	59		
		C	41		

*TNFRII *	−1709 A/T, rs652625	AA	90.7	136	0.548
		AT	9.3	14	
		TT	—	—	
		A	95		
		T	5		

*TNFRII *	−3609 C/T, rs590368	CC	35.3	53	0.45
		CT	50.7	76	
		TT	14	21	
		C	61		
		T	39		

^a^ *P* value for Hardy-Weinberg equilibrium. SNP: single nucleotide polymorphism; TNFR: tumor necrosis factor receptor; GT: Guanidine/Thymine; GC: Guanidine/Cytosine; AT: Adenine/Thymine; CT: Cytosine/Thymine.

**Table 4:** Frequency of genotypes combinations of *TNFRI* and *TNFRII* in patients with rheumatoid arthritis and healthy individuals.

Genotypes *TNFRI* −609	Genotypes *TNFRII* −3609	Frequency of combination in RA, %, (*n*)	Frequency of combination in control %, (*n*)
GT	CT	25.8 (110)	24.2 (36)
GT	CC	10.6 (45)*	22.1 (33)
GG	CT	22.5 (96)	16.8 (25)
GG	CC	12.9 (55)	9.4 (14)
TT	CT	7.0 (30)	9.4 (14)
GT	TT	7.3 (31)	6 (9)
GG	TT	6.1 (26)	4.7 (7)
TT	CC	4.9 (21)	4 (6)
TT	TT	2.8 (12)	3.4 (5)

*Statistical significant difference, *P* < 0.05.

## Abbreviations

CD: Cluster of differentiation
ELISA: Enzyme-linked immunosorbent assay
IgG: Immunoglobulin G
LPS: Lipopolysaccharide
MFI: Mean fluorescence intensity
PBMC: Peripheral blood mononuclear cell
PCR: Polymerase chain reaction
PE: Phycoerythrin
RFLP: Restriction fragment length polymorphism
SNP: Single nucleotide polymorphism
TAE: Tris-acetate-EDTA
TNF*α*: Tumor necrosis factor alpha
TNFR: Tumor necrosis factor receptor
sTNFR: Soluble tumor necrosis factor receptor.
